# Effect of drying methods on the retention of bioactive compounds in African eggplant

**DOI:** 10.1002/fsn3.623

**Published:** 2018-03-13

**Authors:** Naomi N. Mbondo, Willis O. Owino, Jane Ambuko, Daniel N. Sila

**Affiliations:** ^1^ Department of Food Science and Technology Jomo Kenyatta University of Agriculture and Technology Nairobi Kenya; ^2^ Department of Plant Science and Crop Protection University of Nairobi Nairobi Kenya

**Keywords:** African eggplant, antioxidant capacity, beta‐carotene, drying, lycopene, total phenolics

## Abstract

African eggplants (*Solanum aethiopicum* L.) are a rich source of bioactive compounds and functional constituents that are beneficial to human health. However, the short shelf life of these vegetables can be a major cause of postharvest losses especially during peak harvesting season. Drying is one of the most convenient technologies for the production of shelf stable food products. However, drying can lead to considerable loss of the available bioactive compounds due to thermal degradation depending on the drying method and temperature conditions. This study investigated the effect of four drying methods (solar, oven, vacuum, and freeze) on the retention of total phenolics, beta‐carotene, antioxidant capacity, and lycopene in five African eggplant (*S. aethiopicum*) accessions (sangawili, manyire green, S00047A, AB2, and aubergine blanche). Samples were dried up to ~10% moisture content. The fresh and dried samples were analyzed for total phenolic content, antioxidant capacity, beta‐carotene content, and the lycopene content. In the fresh state, beta‐carotene, total phenolic content, and free radical scavenging activity ranged between 14.75 ± 0.50 and 29.50 ± 0.77 mg/100 g db, 751.21 ± 1.73 and 1,363.95 ± 2.56 mg/100 g GAE db, and 99.58 and 325.61 mg/ml db IC_50_ value, respectively. The accession S00047 showed highest total phenolic content and lowest IC_50_ value in the fresh samples. The results also showed that total phenolic content, antioxidant capacity, and beta‐carotene contents were significantly (*p* < .05) affected by drying method and drying temperature with freeze‐drying presenting the highest retention. Overall, 36.26%s–95.05% (total phenolics) and 31.44%–99.27% (beta‐carotene) were retained during freeze‐drying. Lycopene was only detected in the dried samples of the accession manyire green but absent in all the fresh samples of all the accessions. This study demonstrates that freeze‐drying was the most effective in retaining the highest bioactive compounds in African eggplants.

## 1 INTRODUCTION

Bioactive compounds comprise of vitamins, carotenoids, flavonoids, and other phenolic compounds which are found in fruits and vegetables in appreciable amounts (Minussi et al., 2003; Zhang & Hamauzu, 2004). These phytochemical components are high in antioxidant and antiradical activities that are responsible for reducing the risk of radical‐mediated pathogenesis such as carcinogenesis, atherosclerosis, diabetes, Alzheimer, cataracts, and age‐related functional decline (Atoui, Mansouri, Boskou, & Kefalas, 2005; Stommel & Whitaker, 2003; Zhang & Hamauzu, 2004). They also have a hypo‐lipidemic as well as an antimicrobial action (Lim, 2015). Recently, there has been increase in consumer awareness toward bioactive components and their potential health benefits, leading to preference for foods which contain more functional bioactive compounds. Consequently, food processors are increasingly focusing on food products with higher bioactive compounds and their maximal retention during processing to meet the market trend (Nambi, Gupta, Kumar, & Sharma, 2016).

African eggplants also known as the scarlet eggplant are wild relatives of the common eggplant (*Solanum melongena*) (Schippers, 2000). They belong to the *Solanum* genus and comprise of cultivated species such as the Gboma eggplant (*Solanum macrocarpon* L.), the scarlet eggplant (*Solanum aethiopicum* L.), and *Solanum anguivi*, which are grown mostly in Africa for their fruits and leaves. Both *S. aethiopicum* L. and *S. macrocarporn* L. are native to Africa (Daunay, Lester, & Ano, 2001), whereas common eggplant (*S. melongena)* is of Asian origin (Meyer, Karol, Little, Nee, & Litt, 2012). The scarlet eggplant (*S. aethiopicum* L.) is one of the five most important vegetables of tropical Africa, together with tomato, onion, pepper, and okra (Lester & Seck, 2004; Lim, 2015; Maundu, Achigan‐Dako, & Morimoto, 2009; Schippers, 2000). The scarlet eggplant is a phenotypically diverse species which is subdivided into four cultivar groups (Gilo, Kumba, Shum, and Aculeatum) (Lester & Niakan, 1986). Gilo is the commonly cultivated group in Africa, and together with Kumba, they are used for their fruits, while Kumba and Shum are used for their leaves. Aculeatum group is utilized as an ornamental as well as a rootstock (Daunay, 2008; Lester & Daunay, 2003; Schippers, 2000).

Eggplants are a rich source of phytochemicals including the anthocyanins as well as the phenolic acids (mostly hydroxycinnamic conjugates, with chlorogenic acid as predominant compound) (Daunay, 2008). These substances are substrates for the polyphenol oxidase enzyme whose activity leads to the rapid browning of cut or injured tissues. In addition, they contribute to the fruit organoleptic properties because they generally impart a bitter taste and interfere with other molecules during the cooking process (Daunay et al., 2001).

The health and nutritional benefits of African eggplants have led to their increased demand and hence production. However, increased production is accompanied by increase in postharvest losses due to their perishable nature. Cold chain systems are ideal in preventing the postharvest losses and maintain quality of perishable commodities. However, these facilities are inadequate or poorly established in developing countries such as Kenya. Furthermore, perishable commodities such as African eggplants are sensitive to chilling injury (Yahia, Barry‐Ryan, & Dris, 2004). Due to the relatively short postharvest life in fresh form, vegetables can be converted to shelf stable forms through processing (Vincente, Manganaris, Ortiz, Sozzi, & Crisosto, 2014). However, processing can induce negative changes in the physical and chemical properties of the product in question (Muthukumarappan & Tiwari, 2010). One of the most commonly used processing methods is drying (Swanson & McCurdy, 2009). Drying can plausibly cause damage to the inherent nutrients and bioactive compounds depending on the drying method and treatment conditions. Therefore, the choice of the drying method and optimization of the drying process are important for bioactive compound retention (Akdaş & Başlar, 2015). Several studies have described the drying characteristics of various vegetable products such as tomato (Kingsly, Singh, Goyal, & Singh, 2007; Movagharnejad & Nikzad, 2007; Mwende, Owino, & Imathiu, 2018), red chilli (Hossain, Woods, & Bala, 2007), sweet pepper (Vengaiah & Pandey, 2007), okra (Doymaz, 2005), carrot (Zielinska & Markowski, 2007), and common eggplant (Doymaz & Göl, 2011). However, information on drying of African eggplant is scanty. To our knowledge, no studies have been devoted to determine the effect of different drying methods on the retention of bioactive compounds in African eggplant.

Hence, the objective of this study was to evaluate the retention of beneficial bioactive compounds, namely total phenolic content, antioxidant capacity, lycopene, and beta‐carotene under four drying methods, namely solar‐drying, oven‐drying, vacuum‐drying, and freeze‐drying in five African eggplant accessions.

## 2 MATERIALS AND METHODS

### 2.1 Plant material

**Figure 1:**
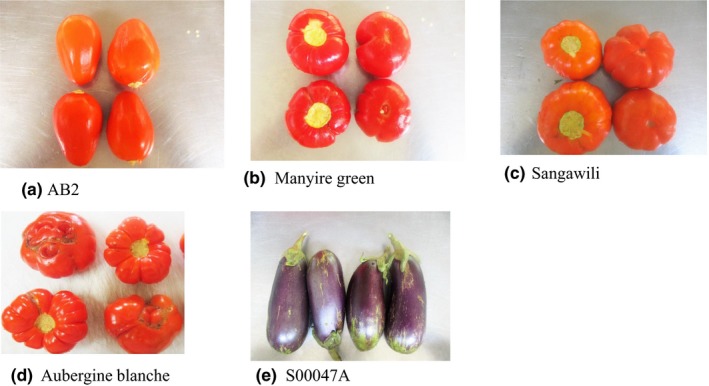
Representative eggplant fruits accessions that were evaluated

Five selected African eggplant accessions with different characteristics were used in this study (Table 1 and Figure 1). These accessions were obtained from the African Vegetable Research and Development Center (AVRDC), Arusha, Tanzania. The accessions were chosen on the basis of size and survival rate in the open‐air field. The accessions with small‐sized fruits were avoided because large quantities would have been required for drying. The selected accessions included the following: AB2, manyire green, sangawili, aubergine blanche, and S00047A. Sixteen plants of each accession were grown in three replicates in a randomized complete block design during the month of May 2016, in an open‐air field plot at the Jomo Kenyatta University of Agriculture and Technology (Juja, Kenya), experimental research farm. Plants were spaced 75 cm by 75 cm between and within the rows and irrigated. Appropriate fertilization was carried out to ensure growth of healthy plants. During transplanting, well‐decomposed manure was mixed with the soil before placing the seedlings. The first basal application of NPK (17:17:17) at a rate of 5 g/plant was performed 2 weeks after transplanting the seedlings, while the second basal application was performed at the flowering stage using the same application rate.

**Table 1:** Accessions that were used and their physical characteristics

Accession registration code used at AVRDC	Genus and species	Accession name	Fruit shape	Color at mature red/stage 2
RV100380	*Solanum aethiopicum*	AB2	Oval	Red
RV100161	*S. aethiopicum*	Manyire green	Flattened and ribbed	Red
RVI00333	*S. aethiopicum*	Sangawili	Spherical and lightly ribbed	Red
RVI00327	*S. aethiopicum*	Aubergine blanche	Flattened and ribbed	Red
RV100455	*Solanum* spp.	S00047A	Semi‐long	Pale purple

### 2.2 Harvesting and sample preparation

African eggplant fruits were harvested at mature red stage. They were stored overnight at a room temperature of 20–23°C and 28%–42% of relative humidity to dissipate the field heat. At the start of each experiment, manyire green, AB2, sangawili, and aubergine blanche were washed in water, allowed to dry, and cut longitudinally into equal quarters. S00047A was cut into slices having the dimensions of 0.5 cm thickness and 2 cm diameter. The cutting/slicing procedure was the same for all the drying experiments. The slices were subjected to drying using the four different methods. Desired temperature conditions inside the drying chambers for oven‐ and vacuum‐drying were obtained for at least 1 hr before each experiment. The sample size was kept constant at 300 ± 0.5 g for all the drying experiments.

### 2.3 Drying processes

#### 2.3.1 Oven‐drying

The drying experiment was carried out at three temperatures (50, 60, and 70°C) in an oven‐drier (Memmert UF 110 model; Memmert GmbH + Co. KG, Schwabach, Germany) with a constant air‐flow rate of 2 m/s. The African eggplant slices were spread in rectangular chambers of 45 cm length by 30 cm width in single‐layer drying. The drying temperatures were chosen on the basis of low, moderate, and high drying temperatures.

#### 2.3.2 Vacuum‐drying

A vacuum‐drier **(**VDO‐4SO model; Mitamura Riken Kogyo Inc., Tokyo, Japan) was used at 50, 60, and 70°C temperature and 60 mbar pressure conditions. Below 60 mbar pressure was too low for drying. The African eggplant slices were spread in square chambers of 30 cm width and length in single‐layer drying.

#### 2.3.3 Freeze‐drying

This was performed using a small‐scale freeze‐drier **(**Alpha1‐4 LD plus‐Martin Christ Model‐101541; Germany). Samples slices were placed in airtight ziplock bags and frozen in a deep freezer at −21°C for 72 hr. The ziplock bags were pierced with several holes and placed in the freeze‐drier. The holes allowed good balance of pressure and temperature inside and outside the ziplock bags during drying. Initial drying was carried out at −41°C and 0.11 mbar, while final drying was carried out at −47°C and 0.055 mbar. In total, the freeze‐drying was carried out for a period of 72 hr. The temperature and pressure conditions used are a recommendation by the drier manufacturer.

#### 2.3.4 Solar‐drying

A small‐scale solar‐drier was used in this experiment. The African eggplant slices were spread in rectangular chambers of 60 cm length by 40 cm width in single‐layer drying. The main structure measured 185 cm wide, 273 cm long, and 255 cm high. The top part of this structure was semicircular in shape with a radius of 50 cm and was entirely covered with a polyvinyl chloride (PVC) material. The dimensions of the door were 60 cm wide and 180 cm high. The PVC material was preferred because it filters radiations such as ultraviolet, which can destroy light‐sensitive nutrients in the material being dried (Leon, Kumar, & Bhattacharya, 2002).

The drying of eggplants was finalized when the moisture content decreased to ~10%. At the end of each drying experiment, moisture content of the samples was determined and the dried samples were stored in ziplock bags at −20°C away from light until further analysis. All the experiments were carried out in three replicates and the results expressed on dry weight basis (db).

### 2.4 Determination of moisture content

The moisture content was determined according to method 984.25 (AOAC, 2005).

### 2.5 Determination of beta‐carotene

Five grams of each of the fresh and dried samples was weighed, and approximately 1.5 g of celite was added together with 10 ml of cold acetone. The mixture was ground in a mortar and pestle and transferred into 50‐ml volumetric flask using a glass funnel plugged with cotton wool. The residual was filtered and washed with cold acetone until devoid of color. Fifteen milliliters of petroleum spirit was dispensed into a separating funnel and the acetone extract slowly added followed by distilled water to eliminate residual acetone. The two phases were allowed to separate, and the lower aqueous layer was carefully removed and discarded. The petroleum spirit fraction containing carotenoids was collected into a conical flask through a funnel having anhydrous sodium sulfate (Na_2_SO_4_) to dry the layer and topped up to 50 ml with petroleum spirit. Beta‐carotene was determined at 440 nm using UV–vis spectrophotometer (UV mini 1240 model; Shimadzu Corp., Kyoto, Japan). The absorbance of standard solutions was used to generate the standard curves (Rodriguez‐Amaya & Kimura, 2004).

### 2.6 Extraction of antioxidants and total phenols

Extracts were prepared according to Wojdyło, Oszmiański, and Czemerys (2007) with a few modifications. Five grams of samples was weighed into amber‐colored bottles containing 50 ml of analytical grade methanol and vortexed for 3 hr. The solution was incubated in darkness for 48–72 hr at room temperature. The extracts were centrifuged for 10 min at 13,000×*g*/relative centrifugal force (RCF) and supernatants used to determine the total phenolic content and antioxidant capacity.

#### 2.6.1 Determination of total phenolic content

Total phenolic content (TPC) was determined by the Folin–Ciocalteu colorimetric method (Wojdyło et al., 2007) with gallic acid as the standard. Two milliliters of 10% (v/v) Folin–Ciocalteu reagent and 4 ml of 0.7 mol/L sodium carbonate were added onto 1 ml of prepared sample extract. The mixture was vortexed and allowed to stand at room temperature for 2 hr. The absorbance was measured at 765 nm using spectrophotometer (Shimadzu UV–1240), and results were expressed as gallic acid equivalent (GAE), milligrams per 100 g of dry matter (db). A standard curve was generated using the absorbances of the gallic acid standards.

#### 2.6.2 Determination of antioxidant activity by radical scavenging effect of DPPH (2,2‐diphenyl‐1‐picryl hydrazyl)

The antioxidant activity of the extracts and the standard was assessed on the basis of the radical scavenging effect of the stable 1,1‐diphenyl‐2‐picrylhydrazyl (DPPH) free radical activity (Sreenivasan, Ibrahim, Kassim, & Noordin, 2007) with some modifications. One milliliter of sample extract and standard was mixed with 0.5 ml of a 1 mmol/L solution of DPPH and 3 ml of methanol. l‐Ascorbic acid was used as the standard. The solution mixtures were incubated for 5 min, and absorbance was measured using spectrophotometer at 517 nm (Shimadzu UV–1240).

The % inhibition was calculated using the formula given below:

$$\left(\%\right)\;\mathrm{inhibition}\;\mathrm{of}\;\mathrm{DPPH}\;\mathrm{activity}=\left[\frac{\left(A_{0}-A_{1}\right)}{A_{0}}\right]*100$$

where *A*_0_ was the absorbance of the blank and *A*_1_ was the absorbance in the presence of the sample. IC_50_ value was calculated using the dose inhibition curve. IC_50_ values denoted the concentration of sample, which was required to scavenge 50% of DPPH free radicals.

The results were also expressed as ascorbic acid equivalent antioxidant capacity (AEAC) in mg ascorbic acid/100 g of sample in dry basis using the following equation:

$$\mathrm{AEAC}\;\mathrm{mg}\;\mathrm{ascibic}\;\mathrm{acid}/100g=\left[\frac{{\mathrm{IC}}_{50}\left(\mathrm{ascorbic}\;\mathrm{acid}\right)}{{\mathrm{IC}}_{50}\left(\mathrm{sample}\right)}\right]*100,000$$

### 2.7 Determination of lycopene content

Lycopene content was determined using the method suggested by Lin and Chen (2005) with some modifications. About 5 g of crushed eggplant sample was mixed with hexane–acetone–ethanol solution (2:1:1 v/v/v) containing 1% BHT (w/v) in amber‐colored sample bottles. The content was then agitated for 20 min after which 15 ml of distilled water was added to the mixture and agitated for 10 min. The solution was separated into polar and a nonpolar phase using a separating funnel. A 50 ml of the upper hexane layer was collected and 1.5 ml aliquot microfiltered using 0.45‐μl membrane filters. Lycopene was analyzed using a Shimadzu brand HPLC (10A model; Tokyo, Japan) fitted with SPD‐10AV UV–vis detector and a C18 ODS (250 mm*4.6 mm*5 μl) column. The mobile phase contained acetonitrile:methanol:dichloromethane: hexane (40:20:20:20, v/v/v/v) at a flow rate of 1.5 ml/min. Injection volume used was 20 μl, while the detection wavelength for lycopene was 470 nm. The temperature of the oven was maintained at 30 °C. Lycopene in the sample was identified by comparing the retention time of pure lycopene from Sigma‐Aldrich.

### 2.8 Statistical analysis

Comparisons among the various accessions and effect of drying method on the dependable variables (beta‐carotene, total phenolics, lycopene, and antioxidant activity) were determined by ANOVA using Stata version 12 software (Stata Corp., College Station, TX, USA), while mean variations were performed using Tukey test at 0.05 significance level.

## 3 RESULTS

### 3.1 Total phenolics, beta‐carotene, antioxidant activity, and lycopene content for fresh fruits

Total phenolic content in the five African eggplant accessions had highly significant differences which ranged between 751.21 mg/100 g (manyire green) and 1,363.95 mg/100 g (S00047A) as shown in Table 2. Statistically, AB2 was insignificantly different from sangawili (*p *=* *.650).

**Table 2:** Total phenolics, beta‐carotene content, IC_50_ values, ascorbic equivalent antioxidant capacity (AEAC), and lycopene content in five African eggplant accessions studied

Accession	Total phenolics (mg/100 g GAE db)	Beta‐carotene (mg/100 g db)	IC_50_ value (mg/ml db)	AEAC mg/100 g	Lycopene mg/100 g
Sangawili	813.77 ± 5.15^b^	14.75 ± 0.50^a^	325.61	307.12	—
Manyire green	751.21 ± 1.73^a^	29.50 ± 0.77^d^	163.28	612.43	—
S00047A	1,363.95 ± 2.56^d^	19.72 ± 0.86^b^	99.58	1,004.21	—
AB2	823.01 ± 3.20^b^	23.77 ± 1.32^c^	283.32	352.96	—
Aubergine blanche	898.82 ± 8.20^c^	14.99 ± 0.46^a^	298.01	335.56	—
Mean	930.17 ± 59.33	20.55 ± 1.52			
CV (%)	24.7	28.7			

For accession mean values represent average ± SE of three replicates and for maturity stage mean value represent average ± SE of accession means. Values with different letters within a column indicate significant differences based on a Tukey test at a level of significance of *p* < .05 (*n* = 3).

The beta‐carotene content for the fresh fruits had an average value of 20.55 mg/100 g db. Manyire green showed the highest beta‐carotene content (29.50 mg/100 g db) (Table 2). On the other hand, aubergine blanche was found not to be statistically different from sangawili (*p *=* *.993).

**Figure 2:**
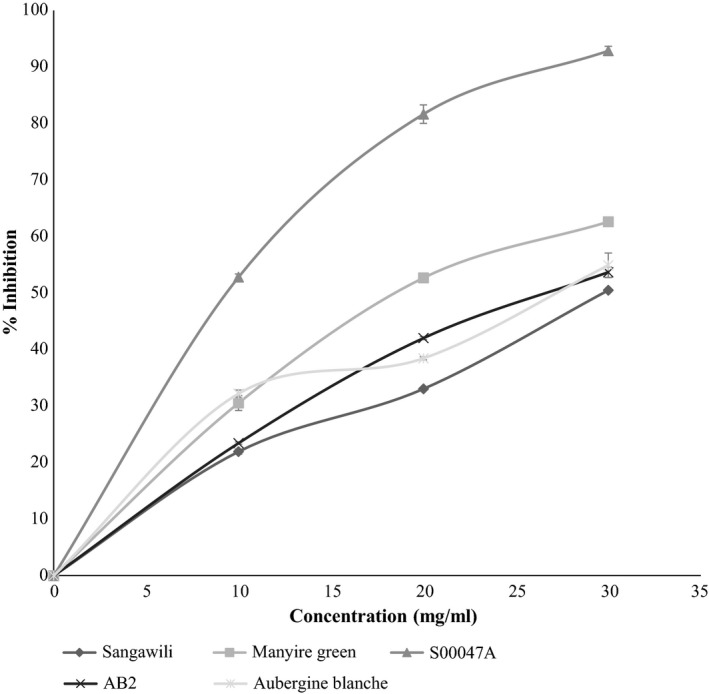
A graph of percentage inhibition against concentration for fresh fruit samples of five African eggplant accessions. Each point is a representation of the mean of three replicates. Standard errors are represented in the figure by the error bars attached to each point

Antioxidant activity was determined in terms of IC_50_ value which means the concentration of a sample that induces 50% inhibition of DPPH free radicals (Karaman et al., 2014). The IC_50_ values for all the five accessions had hyper variability ranging between 99.58 mg/ml db (S00047A) and 325.61 mg/ml db (sangawili) (Table 2). The % inhibition increased with increase in concentration for all the accessions, with S00047A having the highest rate of increase (Figure 2) Notably, the accession S00047A which had the highest total phenolic content showed the lowest IC_50_ value. The AEAC was highest in S00047A (10,004.21 mg/100 g db) and lowest in sangawiili (307.12 mg/100 g db) (Table 2). No lycopene was detected in the fresh fruits in all the African accessions analyzed.

### 3.2 Effect of drying method on the retention of total phenolics, beta‐carotene, antioxidant activity, and lycopene

The four different drying methods resulted in drastic weight losses of ~90%. Drying also resulted in considerable shrinkage of the vegetable slices resulting in crispiness. Drying method resulted in significant changes (*p *=* *.001) for each dependent variable (total phenols, beta‐carotene, and IC_50_ value) for all the five accessions. The effect of drying method was determined in each accession independently. The highest retention rate of total phenolics was observed in freeze‐dried samples (95.05%) followed by oven‐drying at 70°C (94.20%) and then vacuum‐drying at 70°C (90.53%). The highest retention of total phenolics was observed in the manyire green accession. On the other hand, the lowest retention of total phenolics was observed in oven‐dried samples at 60°C with 33.69% for AB2 accession (Table 3). Freeze‐drying and oven‐drying at 70°C did not have a significant effect on the degradation of total phenolics in manyire green and aubergine blanche (Table 3). With regard to beta‐carotene, significantly highest retention rate was observed after freeze‐drying in manyire green (29.28 mg/100 g db) where 99.27% of beta‐carotene was retained (Table 3). The free radical scavenging activity with respect to the IC_50_ values significantly declined in the four drying methods compared to the fresh state. This corresponded to a rise in the AEAC (Table 4). Notably, lycopene was detected in low quantities in only one dried accession (manyire green) while absent in the rest (Table 3). In addition, there was no significant difference between the effect of freeze‐drying and vacuum‐drying on the lycopene content.

**Table 3:** Total phenolic content (mg/100 g GAE db) and beta‐carotene content (mg/100 g db) for five accessions (sangawili, manyire green, S00047A, AB2, and aubergine blanche) after drying

Accession drying method	Sangawili		Manyire green		Lycopene	S00047A		AB2		Aubergine blanche	
	Total phenols	β‐Carotene	Total phenols	β‐Carotene		Total phenols	β‐Carotene	Total phenols	β‐Carotene	Total phenols	β‐Carotene
Fresh	813.77^j^	14.75^e^	751.21^g^	29.50^f^	—	1,363.95^h^	19.72^d^	823.01^i^	23.77^f^	898.82^g^	14.99 ^g^
FD	595.80^e^	14.61^e^	714.01^f^	29.28^f^	0.17^b^	494.60^a^	6.20^c^	657.27^h^	13.98^e^	574.31^de^	14.88 ^g^
SD	560.58^d^	7.03^cd^	422.57^a^	10.91^b^	0.15^a^	547.08^b^	0.73^a^	628.32^g^	12.08^de^	447.32^a^	12.58^f^
OD70	761.05^i^	1.53^a^	707.66^f^	3.63^a^	0.16^ab^	1,024.60^g^	3.68^b^	587.53^f^	11.42^d^	585.21^e^	2.10^a^
OD60	467.73^a^	2.58^a^	487.86^b^	2.25^a^	0.16^ab^	748.97^e^	3.27^b^	277.29^a^	2.58^a^	574.67^de^	6.27^bc^
OD50	670.38^h^	5.55^b^	574.27^d^	9.76^b^	0.17^b^	682.44^d^	5.40^c^	362.70^b^	5.31^ab^	556.94^d^	5.42^b^
VD70	621.00 ^g^	8.07^d^	680.07^e^	27.12^e^	0.17^b^	993.22^f^	6.26^c^	548.86^e^	8.27^c^	646.46^f^	8.63^e^
VD 60	529.55^c^	6.42b^c^	557.10^c^	20.83^d^	0.17^b^	639.95^c^	6.30^c^	465.20^c^	6.78^bc^	533.85^c^	7.12^c^
VD50	493.09^b^	5.50^b^	564.83^cd^	19.14^c^	0.17^b^	644.86^c^	5.60^c^	525.15^d^	6.11^bc^	481.30^b^	6.15^bc^
Mean	612.55	7.34	606.62	16.98	0.17	793.3	6.35	541.71	10.03	588.76	8.68
SE±	21.8	0.86	20.81	1.99	0.01	51.84	0.99	29.98	1.17	24.08	0.83

FD, freeze‐drying; SD, solar‐dying; OD70, oven‐drying at 70°C; OD60, oven‐drying at 60°C; OD50, oven‐drying at 50°C; VD70, vacuum‐drying at 70°C; VD60, vacuum‐drying at 60°C; VD50, vacuum‐drying at 50°C. Values with different letters within a column indicate significant differences based on a Tukey test at a level of significance of *p* < .05 (*n* = 3).

**Table 4:** IC_50_ values (mg/ml db) and ascorbic equivalent antioxidant capacity (AEAC) (mg/100 g) for five accessions (sangawili, manyire green, S00047A, AB2, and aubergine blanche) after drying

Accession	Sangawili		Manyire green		S00047A		AB2		Aubergine blanche	
Drying method	IC_50_ value	AEAC	IC_50_ value	AEAC	IC_50_ value	AEAC	IC_50_ value	AEAC	IC_50_ value	AEAC
Fresh	325.61	307.12	163.28	612.44	99.58	1,004.22	283.32	352.96	298.01	335.56
FD	5.70	17,543.86	4.98	20,080.32	4.25	23,529.41	6.58	15,197.57	6.37	15,698.59
SD	5.47	18,281.54	5.78	17,301.04	4.80	20,833.33	5.49	18,214.94	5.16	19,379.84
OD70	16.52	6,053.27	6.76	14,792.90	4.73	21,141.65	15.19	6,583.28	5.57	17,953.32
OD60	18.89	5,293.81	5.73	17,452.01	4.72	21,186.44	13.53	7,390.98	5.27	18,975.33
OD50	10.11	9,891.20	5.62	17,793.59	4.90	20,408.16	10.31	9,699.32	5.59	17,889.09
VD70	4.85	20,618.56	5.38	18,587.36	4.79	20,876.83	9.35	10,695.19	5.99	16,694.49
VD 60	5.86	17,064.85	5.07	19,723.87	5.08	19,685.04	11.90	8,403.36	11.08	9,025.27
VD50	7.24	13,812.15	8.97	11,148.27	4.97	20,120.72	11.73	8,525.15	13.47	7,423.90

FD, freeze‐drying; SD, solar‐dying; OD70, oven‐drying at 70°C; OD60, oven‐drying at 60°C; OD50, oven‐drying at 50°C; VD70, vacuum‐drying at 70°C; VD60, vacuum‐drying at 60°C; VD50, vacuum‐drying at 50°C. IC_50_ values derived from the dose inhibition curve whose each point is a representation of the mean of three replicates.

### 3.3 Effect of drying temperature on the retention of total phenolics, beta‐carotene, antioxidant activity, and lycopene during oven‐ and vacuum‐drying

The results show that temperature conditions in oven‐ and vacuum‐drying had a significant effect on retention of the bioactive compounds determined in the five accessions (Tables 3 and 4). There was significant decline in the total phenolic content as the drying temperature reduced in oven‐ and vacuum‐drying. The highest retention of 94.20% was observed in oven‐drying at 70°C of manyire green while lowest retention of 33.69% was observed at 60°C in AB2 accession. The percentage retention of total phenolics ranged between 65.11% (Aubergine blanche) and 94.20% (manyire green); 33.69% (AB2) and 64.94% (manyire green); 44.07% (AB2) and 82.38% (sangawili) in oven‐drying at 70, 60, and 50°C, respectively (Table 3). On the other hand, the retention rate ranged between 66.69% (AB2) and 90.53% (manyire green); 46.92% (S00047A) and 74.16% (manyire green); 47.28% (S00047A) and 75.19% (manyire green) in vacuum‐drying at 70, 60, and 50°C respectively (Table 3). With respect to beta‐carotene, decrease in temperature resulted in increase in beta‐carotene content in oven‐drying. The highest retention was observed in vacuum‐drying at 70°C where 91.93% (manyire green) of beta‐carotene was retained. In contrast, lowest retention was 7.63% in manyire green observed after oven‐drying at 60°C (Table 3) The IC_50_ values increased with decrease in temperature from a low of 4.79 mg/ml db (S00047A) at 70°C vacuum‐drying to a high of 13.47 mg/ml db (aubergine blanche) at 50°C vacuum‐drying. An increase in IC_50_ value corresponded to a decrease in the AEAC content and vice versa (Table 4). The results showed no significant effect of temperature on the lycopene content in dried manyire green (Table 3).

## 4 DISCUSSION

The results of this study indicate that the five African eggplant accessions are high in bioactive compounds as exhibited by high total phenolic content in particular. The significant differences in the total phenolic content between the five accessions as shown in Table 2 may be attributed to the differences in genetic makeup of the accessions, which is one of the influencing factors in the synthesis of phenolic compounds in plants (Hanson et al., 2004). The accession S00047A had the highest phenolic content in fresh samples. This may be attributed to its larger surface area due to its semi‐long shape. Studies have shown that most phenolic compounds are concentrated in the skin surface of fruits (Dadalı, Kılıç Apar, & Özbek, 2007). The total phenolic content of the five accessions in fresh samples is of similar range in different Turkish eggplant *(S. melongena)* cultivars reported by Okmen et al. (2009) and Hanson et al. (2006). Significant differences in beta‐carotene content were observed between the five accessions. This may be similarly attributed to the differences in their genetic makeup (Rodriguez‐Amaya & Kimura, 2004). Manyire green which had highest beta‐carotene content has a deep red color, different from the light red and purple color of the other accessions as shown in Figure 1. Carotenoids are responsible for impacting fruits with a yellow to red color. In addition, manyire green plant is characterized by a short canopy which could mean greater exposure to sunlight and subsequent increase in carotenogenesis (Rodriguez‐Amaya & Kimura, 2004). The beta‐carotene content of manyire green (29 mg/100 g db) is comparable to tomato as reported by Mwende et al. (2018). On the other hand, the beta‐carotene content reported in this study (Table 2) is higher compared to previous reports by Chepngeno, Owino, Kinyuru, and Nenguwo (2016) and Msogoya, Majubwa, and Maerere (2014) on African eggplants. This could suggest a wide variability in the beta‐carotene content among African eggplant accessions (Plazas et al., 2014). With respect to the antioxidant capacity, the results showed a significant (*p *=* *.001) positive correlation (*r* = .822) between the total phenolic content and the AEAC. Interestingly, it was observed that the higher the total phenolic content, the lower the IC_50_ value and the higher the AEAC (Table 2). This is critical and may be attributed to the fact that the phenolic compounds are effective hydrogen donors and thus good antioxidants (Desai et al., 2013). Similar positive correlations between phenolic content and antioxidant capacity have been reported by Hanson et al. (2006) and Okmen et al. (2009) in eggplants (*S. melongena*). On the other hand, the correlation between the beta‐carotene content and the AEAC was insignificant. Most studies on antioxidant capacity in eggplants used Trolox equivalent antioxidant capacity (TEAC) assay or radical cation 2,2‐azinobis (3‐ethylbenzothiazoline‐6‐sulfonate) (ABTS) assay (Okmen et al., 2009; Zaro et al., 2015). However, both the ABTS and DPPH assays are based on radical scavenging activity of antioxidants toward the two free radicals and have been widely applied in fruits and vegetables because they are simple and quick to perform (Grigelmo‐Miguel, Rojas‐Graü, Soliva‐Fortuny, & Martın‐Belloso, 2009).

The effect of drying method as presented in Table 3 shows that freeze‐drying was the most effective in retaining the optimal total phenolic contents and beta‐carotene contents. This is in comparison with solar‐drying, vacuum‐drying, and oven‐drying and may be attributed to the gentle process of lyophilization whereby enzymatic, bacterial, and chemical changes are largely avoided. Despite the effectiveness of freeze‐drying, this study showed slight degradation of total phenolics and beta‐carotene. Phenolic compounds decline after freeze‐drying may be associated with cellular decompartmentalization during prefreezing step (Chang, Lin, Chang, & Liu, 2006) followed by the reaction of phenolics with proteins in the dehydration process, which could subsequently affect their extractability (Martín‐Cabrejas et al., 2009). Similar degradation of phenolic compounds has been reported by Zaro et al. (2015), where a marked drop of antioxidants in eggplant fruit was observed during freeze‐drying. In contrast, solar‐drying and oven‐drying resulted in least retention of beta‐carotene (3.70%) and total phenolics (33.6%), respectively. Loss of total phenolics during solar‐drying and oven‐drying (60, 50°C) may be attributed to enzymatic processes by polyphenol oxidases (Lim & Murtijaya, 2007). In addition, solar‐drying is dependent on the weather conditions which contribute to uneven losses (Lim & Murtijaya, 2007). Extended drying periods have also been shown in some cases to lead to higher losses in nonblanched tissues due to enzymatic browning (Kerkhofs, Lister, & Savage, 2005; McSweeney & Seetharaman, 2015). This may explain the low retention of bioactive compounds caused by solar‐drying where the period of drying is longer depending on the weather conditions. In this study, all the four drying methods used resulted in significant decline in the bioactive compounds. This is in agreement with Zaro et al. (2015) who reported on chlorogenic acid retention in white and purple eggplant after processing and cooking where it was observed that high losses (80%–98%) of TEAC (Trolox equivalent antioxidant capacity) and CQA (5‐O‐caffeoyl‐quinic acid) content occurred regardless of the drying method.

The detection of lycopene in the dried manyire green samples and its absence in the fresh samples may be associated with greater extractability of carotenoids from processed samples (Rodriguez‐Amaya & Kimura, 2004). In addition, lycopene bioavailability has been reported to exhibit an increase in heat‐processed tomatoes compared with unprocessed tomatoes (van het Hof et al., 2000). Among the five accessions used in this study, only the dried samples of manyire green were found to contain lycopene. This may be of interest because the red color of its peel is similar to that of tomato and as ripening progressed, the red color deepened and developed into the pulp. In tomato berries, the lycopene concentration increases with maturation leading to the development of red color (Kirk & Tilney‐Bassett, 1978).

The effect of drying temperature during oven‐ and vacuum‐drying as presented in Table 3 shows that temperature had a significant effect on the retention of the total phenolics and beta‐carotene contents. The retention rate of total phenolics decreased with decrease in temperature. This observation may be associated with the immediate inactivation of polyphenol oxidase enzymes at 70°C and delayed inactivation at 60 and 50°C. Higher drying temperatures inactivate or at least inhibit polyphenol oxidase (PPO)‐mediated oxidation of phenolics (Lim & Murtijaya, 2007). Zaro et al. (2015) reported that drying at 50°C caused greater losses of phenolic antioxidants than at 70°C. In addition, high thermal treatment and/or extended periods of drying may be responsible for a significant decline in natural antioxidants, as most of these compounds are relatively unstable. This study also showed that vacuum‐drying at 70°C led to higher degradation of phenols as compared to oven‐drying at 70°C. Similar results were reported in the drying of mandarin slices by Akdaş and Başlar (2015). At 60 and 50°C, the opposite was observed which could be associated with longer drying period for oven‐drying in comparison to vacuum‐drying. The phenolic content degrades because of thermal degradation during the drying process while volatile and semivolatile phenolic compounds can evaporate with water in the samples during vacuum‐drying (Akdaş & Başlar, 2015). Antioxidant capacity degradation increased as the drying temperature decreased, and this concurs with Karaman et al. (2014), who also reported that vacuum‐drying prevented antioxidant capacity degradation more than oven‐drying.

Contrastingly, higher drying temperature resulted in significantly low retention of beta‐carotene while lower temperatures led to significantly higher retention (Table 3). This may be attributed to higher rate of isomerization and oxidation at 70°C as compared to 60 and 50°C (Eldahshan & Singab, 2013). Similar observation was reported by Mwende et al. (2018) and Demiray, Tulek, and Yilmaz (2013) in the drying of tomatoes. On the other hand, carotenoid retention has been shown to decrease with longer processing time, higher processing temperature, and cutting or puréeing of the food regardless of the processing method. Retention is significantly improved by reducing the processing time, lowering the temperature, and shortening the time lag between peeling, cutting, or puréeing and processing (Rodriguez‐Amaya & Kimura, 2004). In some cases however, processing leads to little or no change to the content and activity of naturally occurring antioxidants, such as lycopene which has been found to be very heat stable (Nicoli, Anese, & Parpinel, 1999). This may support the results of this study whereby there was insignificant difference in the lycopene content regardless of the drying method or temperature in manyire green (Table 3).

## 5 CONCLUSION

This study indicates that each accession was affected by drying method uniquely perhaps due to differences in internal structures and genetic makeup. Overall, the four drying methods resulted in the decline in total phenolic content, beta‐carotene content and antioxidant capacity as compared to the fresh samples. However, freeze‐drying significantly best retained the total phenolic and beta‐carotene contents compared to oven‐drying, vacuum‐drying, and solar‐drying. Drying improved the extractability of lycopene in the manyire green accession and hence its detection. Considering that choice of drying method depends on various factors such as the type of product, availability of dryer, energy consumption, cost of dehydration, and quality of dehydrated product; these results may be a demonstration of the potentiality of optimizing drying technology to reduce postharvest losses in African eggplants and thus combat the problem of hunger during the drought periods when fresh fruits and vegetables are scarce.

## CONFLICT OF INTEREST

None declared.
